# Structural Racism and Adolescent Mental Health Disparities in Northern California

**DOI:** 10.1001/jamanetworkopen.2023.29825

**Published:** 2023-08-18

**Authors:** Julia Acker, Sara Aghaee, Mahasin Mujahid, Julianna Deardorff, Ai Kubo

**Affiliations:** 1 School of Public Health, University of California, Berkeley; 2 Kaiser Permanente Northern California Division of Research, Oakland

## Abstract

**Importance:**

Understanding how structural racism is associated with adolescent mental health is critical to advance health equity.

**Objective:**

To assess associations between neighborhood privilege, measured by the Index of Concentration at the Extremes (ICE) and adolescent depressive symptoms, suicidality, and related racial and ethnic disparities.

**Design, Setting, and Participants:**

This was a retrospective cohort study using electronic health records of adolescents aged 12 to 16 years who attended well-teen visits between 2017 and 2021. Kaiser Permanente Northern California is an integrated health care delivery system serving 4.6 million members. The cohort included 34 252 individuals born singleton at an affiliated facility from January 1, 2005, to December 31, 2009, and who had completed at least 1 mental health screener during a well-teen visit by November 23, 2021.

**Exposures:**

American Community Survey 2016 to 2021 5-year estimates were used to calculate ICE scores for adolescents’ residential census tract at ages 10 to 11. Three ICE measures were used as proxies of structural racism: racial privilege (ICE–race and ethnicity; hereinafter ICE–race), economic privilege (ICE–income), and combined economic and racial privilege (ICE–income plus race and ethnicity; herinafter ICE–income plus race). ICE scores were categorized into quintiles based on California statewide distributions.

**Main Outcomes and Measures:**

Depressive symptoms and suicidality were assessed through self-report screeners during well-teen visits. Depressive symptoms were considered to be present if patients had a score on the Patient Health Questionnaire-2 of 3 or higher (the tool uses a Likert scale to determine the frequency [0 = not at all; 3 = nearly every day] that they had depressed mood and lack of pleasure in usual activities in the past 2 weeks; responses were summed and dichotomized).

**Results:**

Analyses included 34 252 adolescents (12-16 years of age; mean [SD] age, 13.7 [0.8] years; 17 557 [51.3%] male, 7284 [21.3%] Asian or Pacific Islander, 2587 [7.6%] Black], 9061 [26.5%] Hispanic, 75 [0.2%] American Indian or Indigenous, 12 176 [35.5%] White, and 3069 [9%] other or unknown). Risks of depressive symptoms and suicidality generally increased with each level of declining neighborhood privilege. Adjusted risk ratios comparing adolescents from neighborhoods with the least to most racial and economic privilege were 1.37 (95% CI, 1.20-1.55) for depressive symptoms and 1.59 (95% CI, 1.23-2.05) for suicidality. Racial disparities between Black and White youth and Hispanic and White youth decreased after adjusting for each ICE measure, and became nonsignificant in models adjusting for ICE–race and ICE–income plus race.

**Conclusions and Relevance:**

In this cohort study, lower neighborhood privilege was associated with greater risks of adolescent depressive symptoms and suicidality. Furthermore, adjusting for neighborhood privilege reduced mental health disparities affecting Black and Hispanic adolescents. These findings suggest that efforts to promote equity in adolescent mental health should extend beyond the clinical setting and consider the inequitable neighborhood contexts that are shaped by structural racism.

## Key Points

**Question:**

Are neighborhood economic and racial privilege associated with adolescent depressive symptoms, suicidality, and racial and ethnic disparities?

**Findings:**

In this cohort study of 34 252 adolescents aged 12 to 16 years, lower neighborhood privilege was associated with greater risks of depressive symptoms and suicidality independently of individual-level sociodemographic characteristics. Additionally, adjusting for neighborhood privilege was associated with reduced mental health disparities affecting Black and Hispanic adolescents.

**Meaning:**

The findings suggest that inequitable neighborhood contexts shaped by structural racism contribute to disparities in adolescent mental health.

## Teaser

This cohort study of data from a large health system in northern California assesses whether neighborhood economic and racial privilege are associated with adolescent depressive symptoms, suicidality, and racial and ethnic disparities.

## Introduction

Adolescent mental health is a pressing public health concern in the US.^1,2^ Recent estimates suggest that 1 in 5 adolescents aged 13-18 years meets diagnostic criteria for a mental health disorder.^3^ The past 2 decades have witnessed a significant rise in rates of adolescent depression and suicide.^4,5,6^ Concurrently, racial and ethnic disparities in both have emerged.^4,5,6,7,8,9^ Suicide rates among Asian or Pacific Islander and Black individuals aged 15-24 years increased by 40% and 47%, respectively, from 2013 to 2019, in contrast to the declining rates among American Indian or Indigenous and White youth.^9^ Our understanding of factors that may be associated with these disparities is limited.^6,10,11^ Children from racially and ethnically minoritized groups remain underrepresented in mental health research, and race and ethnicity are often omitted as analytic variables.^10,11,12^ Few mental health studies have incorporated measures of structural racism, which is a fundamental cause of racial and ethnic health inequities.^13,14,15,16^ When adolescent mental health studies do incorporate racism, it is typically confined to examining experiences of interpersonal racial discrimination,^17,18,19^ with less attention to upstream structural inequities that may shape mental health.^20,21^

Historically, structural racism in the US has manifested through institutionalized practices and policies, such as racially restrictive covenants and mortgage redlining, which have shaped and sustained inequitable access to health care, quality education, safe and affordable housing, employment, wealth-building opportunities, and other resources that are critical to well-being.^16^ These inequities reinforce both racial and socioeconomic inequality, creating increasingly unequal developmental environments for children.^22,23^ Structural racism is often measured at the area level (eg, neighborhood) to capture conditions created by historical and ongoing racial residential segregation.^24,25,26,27^ Examples of measures include racial residential segregation (eg, the dissimilarity index), neighborhood ethnic density or racial composition, neighborhood socioeconomic disadvantage (eg, the area deprivation index), and historical redlining.^15,25,26,28^ Studies examining structural racism in relation to physical and mental health have focused primarily on adults and young children.^20,29,30,31^ While research has consistently linked neighborhood socioeconomic disadvantage with poorer mental health,^32^ studies investigating associations between racial residential segregation or ethnic density and mental health have produced mixed results.^29,33^ While the “ethnic density hypothesis” identifies protective features of ethnic enclaves (ie, neighborhoods with a high concentration of a specific racial or ethnic group), such as social support or cohesion and a potential buffer against racial discrimination,^34,35^ a structural racism perspective underscores the pernicious repercussions of geographically entrenched social stratification, a byproduct of structural racism.^14,36^

To our knowledge, no studies of adolescent mental health have used a measure of structural racism that captures both racial and economic inequities. Given that structural racism operates on multiple levels and through various mechanisms, it is important to use multidimensional measures that capture intersections of pathways leading to health disparities.^25,37^ The Index of Concentration at the Extremes (ICE) has been applied in public health research to quantify spatial inequality by race and ethnicity, income, and race and income combined.^38^ Previous studies have linked the ICE to health disparities including adverse birth/pregnancy outcomes,^39,40^ infant mortality,^37,39,40^ cancer,^39^ premature mortality,^37,39^ and hypertension.^39,41^ In this study, we used the ICE to examine associations between structural racism and adolescent depressive symptoms and suicidality, using electronic health records (EHRs) from a large, integrated health care delivery system in northern California. We hypothesized that residence in a less privileged neighborhood as measured by ICE would be associated with greater risk of depressive symptoms and suicidality, independently of individual sociodemographic characteristics. Additionally, we hypothesized that adjustment for ICE would attenuate observed racial and ethnic disparities in the outcomes.

## Methods

### Setting

This retrospective cohort study used EHRs from Kaiser Permanente Northern California (KPNC), an integrated health care delivery system serving 4.6 million members. KPNC members are covered through employer-sponsored insurance, Medicaid, Medicare, and individual plans. The membership is sociodemographically representative of the northern California population with the exception of income, as members slightly underrepresent individuals with very low income (<200% below the federal poverty level).^42,43^ The KPNC Institutional Review Board approved this study with a waiver of the requirement for informed consent because it was a data-only study with no participant contact. This report follows the Strengthening the Reporting of Observational Studies in Epidemiology (STROBE) reporting guideline for cohort studies.

### Participants

This study includes individuals born singleton at an affiliated facility from January 1, 2005, to December 31, 2009, who were screened for depressive symptoms and suicidality at a well-teen primary care appointment at or before November 23, 2021. Adolescents were excluded if they had non-California addresses at ages 10 to 11 years or were missing data on covariates. In multichild families, we excluded noneldest siblings to mitigate family clustering effects.

### Outcomes

Outcomes were the presence of depressive symptoms and suicidality as indicated by any positive screen on the well-teen questionnaire. In our cohort, the number of well-teen visits with a recorded screening ranged from 1 to 5 (mean [SD], 1.38 [0.62] screenings). Depressive symptoms were assessed using the 2-item Patient Health Questionnaire-2 (PHQ-2), which asks respondents to rate on a Likert scale the frequency (0 = “not at all,” 1 = ”several days,” 2 = “more than half the days,” and 3 = “nearly every day”) that they have had depressed mood (“feeling down, depressed, or hopeless”) and anhedonia (“little interest or pleasure in doing things”) in the past 2 weeks. The PHQ-2 scores are calculated by summing responses (range, 0-6). Scores were dichotomized, with a score of 3 or higher indicating the presence of depressive symptoms.^44,45^ A validation study^45^ of the PHQ-2 in adolescents found this cutoff had a sensitivity of 74% and specificity of 75% for detecting major depression. Suicidality was assessed using 1 item: “Have you thought seriously about killing yourself, made a plan, or tried to kill yourself?” (yes/no).

### Exposures

We calculated ICE scores for California census tracts using 2016 to 2021 American Community Survey 5-year estimates of household income, race and ethnicity, and household income by race and ethnicity. Three ICE measures were used as proxies of structural racism: racial privilege (ICE–race and ethnicity; hereinafter ICE–race), economic privilege (ICE–income), and combined racial and economic privilege (ICE–income plus race and ethnicity; herinafter ICE–income plus race).^38^ Scores were categorized into quintiles based on statewide distributions and linked to adolescents’ residential address at ages 10-11.

ICE measures were calculated using the formula *ICE*_i_ = (*A*_i_ − *P*_i_)/*T*_i_, where, for each census tract *i*, *A*_i_ is the number of residents in the privileged group, *P*_i_ is the number of residents in the disadvantaged group, and *T*_i_ is the total population for whom race and ethnicity or income level is known in census tract *i*. ICE scores can range from −1 (100% of the population belongs to the disadvantaged group) to 1 (100% of the population belongs to the privileged group). A score of 0 would signify either an absence of residents from both groups or an equal distribution across them.^46^

The extreme groups for ICE–income correspond to the ACS categories approximating the 20th and 80th percentiles of the 2021 national household income distribution (<$30 000 and ≥$150 000, respectively).^47^ ICE–race and ICE–income plus race set as the extremes non-Hispanic White and non-Hispanic Black individuals, and high-income non-Hispanic White and low-income Black individuals, respectively. The extreme groups were defined this way because Black-White residential segregation is the most extreme and persistent form of US racial segregation and low-income Black vs high-income White persons ‘‘continue to occupy opposite ends of the socioeconomic spectrum’’ in the US.^37,48,49^ While these 2 measures are constructed around the Black-White dichotomy, we expect lower scores on these metrics to negatively affect residents of all racial and ethnic groups given the legacy of pro-White and anti-Black policies and practices that created disparate neighborhood conditions.^16^

### Covariates

Covariates included maternal education level and maternal age at delivery (proxies for socioeconomic status),^50,51^ and adolescent sex and race and ethnicity. Maternal education level was categorized as high school or less, some college, college graduate, or postgraduate education. Maternal age at delivery was continuous. Race and ethnicity were sourced primarily from information collected by clinical staff during hospitalization for the delivery of the baby. We grouped race and ethnicity into 6 categories: Asian or Pacific Islander (non-Hispanic), Black (non-Hispanic), Hispanic (any race), American Indian or Indigenous (non-Hispanic), White (non-Hispanic; reference group), and other (not defined further) or unknown (non-Hispanic). The categories Asian or Pacific Islander and other were prespecified in the database. Due to small sample sizes, American Indian or Indigenous and other or unknown adolescents were combined into 1 group for analyses. Race and ethnicity was included as a covariate given its association with neighborhood privilege and mental health.

### Statistical Analysis

Analyses were conducted using R, version 4.2.2 (R Foundation for Statistical Computing).^52^ Descriptive analysis included distributions of sociodemographic variables by ICE quintiles. To assess the association between the 3 ICE measures and depressive symptoms and suicidality, we estimated unadjusted and adjusted risk ratios (ARRs) and 95% CIs using log-binomial generalized estimating equations (GEEs) with exchangeable working correlation structures and robust standard errors. We used GEEs to examine racial and ethnic differences in the outcomes before and after adjusting for ICE. The GEE estimates account for clustering of adolescents within census tracts.^53^ The threshold for statistical significance was defined by the 95% CIs: a risk ratio was considered significant if the 95% CI did not include 1. This is equivalent to a 2-tailed *P* < .05.

## Results

The analytic cohort included 34 066 adolescents screened for depressive symptoms and 33 581 screened for suicidality (34 252 unique adolescents; 47 177 unique screenings; Figure). The total cohort was 51.3% male (n = 17 557); 12 to 16 years old (at first screening: mean [SD] age, 13.6 [0.8] years); and from 2813 census tracts. The study population was 21.3% Asian or Pacific Islander (n = 7284), 7.6% Black (n = 2587), 26.5% Hispanic (n = 9061), 35.5% White (n = 12 176), and 9.0% other or unknown (n = 3069). Table 1 describes demographic characteristics of the sample overall and by the least- and most-privileged ICE quintiles. For each ICE measure, White adolescents were overrepresented in the most-privileged quintiles and underrepresented in the least-privileged quintiles; the opposite pattern was observed among Black and Hispanic adolescents. Asian or Pacific Islander adolescents were underrepresented in the most-privileged quintile of ICE–race.

**Figure.**
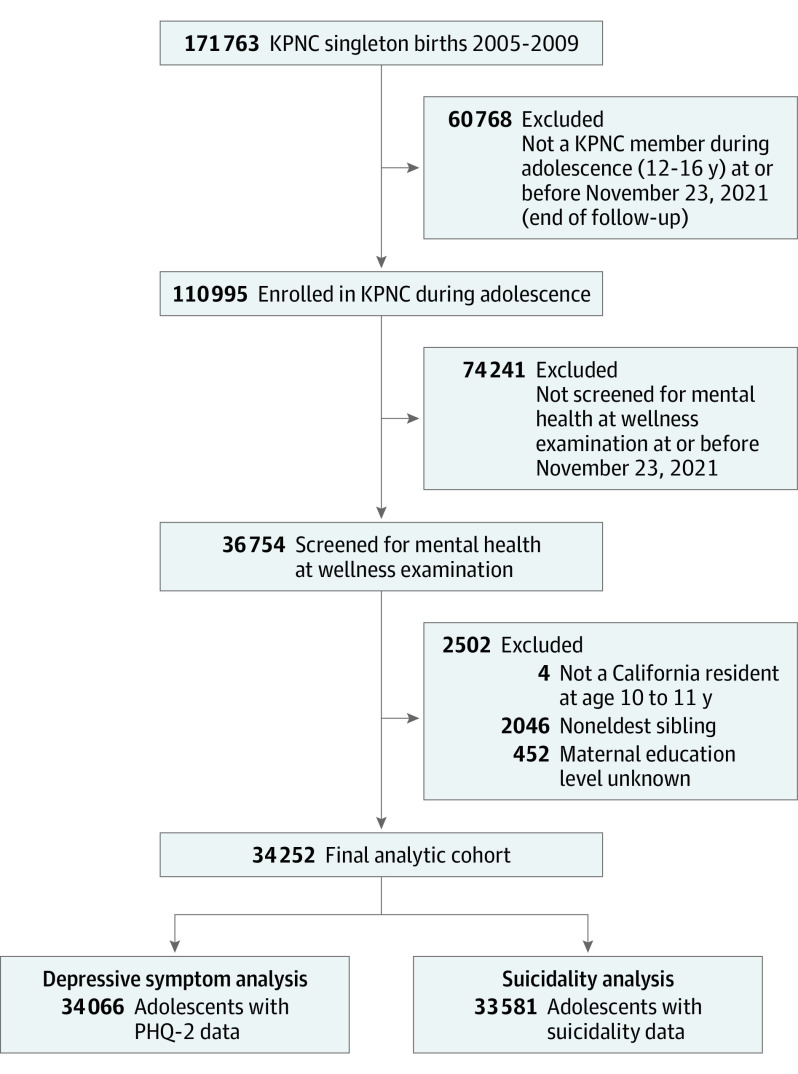
Patient Flowchart KPNC indicates Kaiser Permanente Northern California; PHQ-2, Patient Health Questionnaire-2.

**Table 1.** Characteristics of Participating Health System Members Aged 12 to 16 Years and Their Mothers, Overall and by the Least- and Most-Privileged Quintiles of the Index of Concentration at the Extremes

Characteristic	Patients, total No. (%)	Patients, No. (%)^a^					
		ICE–race and ethnicity		ICE–income		ICE–income plus race and ethnicity	
		Quintile 1 (least privileged)	Quintile 5 (most privileged)	Quintile 1 (least privileged)	Quintile 5 (most privileged)	Quintile 1 (least privileged)	Quintile 5 (most privileged)
Overall	34 252 (100)	5318 (15.5)	4971 (14.5)	2069 (6.0)	13 572 (39.6)	2229 (6.5)	9754 (28.5)
Sex							
Male	17 557 (51.3)	2713 (51.0)	2531 (50.9)	1040 (50.3)	6980 (51.4)	1117 (50.1)	5013 (51.4)
Female	16 695 (48.7)	2605 (49.0)	2440 (49.1)	1029 (49.7)	6592 (48.6)	1112 (49.9)	4741 (48.6)
Race and ethnicity							
Asian or Pacific Islander	7284 (21.3)	1190 (22.4)	461 (9.3)	280 (13.5)	3930 (29.0)	363 (16.3)	1855 (19.0)
Black	2587 (7.6)	967 (18.2)	95 (1.9)	407 (19.7)	499 (3.7)	435 (19.5)	308 (3.2)
Hispanic	9061 (26.5)	2024 (38.1)	707 (14.2)	825 (39.9)	2429 (17.9)	890 (39.9)	1601 (16.4)
American Indian or Indigenous^b^	75 (0.2)	10 (0.2)	16 (0.3)	8 (0.4)	26 (0.2)	7 (0.3)	20 (0.2)
White	12 176 (35.5)	644 (12.1)	3397 (68.3)	393 (19.0)	5412 (39.9)	360 (16.2)	5109 (52.4)
Other, unknown^c^	3069 (9.0)	483 (9.1)	295 (5.9)	156 (7.5)	1276 (9.4)	174 (7.8)	861 (8.8)
Maternal education level							
High school or less	10 208 (29.8)	2460 (46.3)	950 (19.1)	1128 (54.5)	2267 (16.7)	1206 (54.1)	1515 (15.5)
Some college	9988 (29.2)	1728 (32.5)	1287 (25.9)	649 (31.4)	3405 (25.1)	686 (30.8)	2402 (24.6)
College graduate	8586 (25.1)	794 (14.9)	1578 (31.7)	208 (10.1)	4525 (33.3)	246 (11.0)	3295 (33.8)
Postgraduate	5470 (16.0)	336 (6.3)	1156 (23.3)	84 (4.1)	3375 (24.9)	91 (4.1)	2542 (26.1)
Maternal age at delivery, mean (SD) y	30.2 (5.7)	28.6 (6.0)	31.4 (5.4)	27.8 (6.2)	31.5 (5.2)	27.9 (6.1)	31.8 (5.2)

Abbreviation: ICE, Index of Concentration at the Extremes.

^a^ California statewide ICE quintiles measured at the census-tract level.

^b^ American Indian or Indigenous adolescents and those categorized as other or unknown were combined for analysis due to small numbers of American Indian or Indigenous adolescents.

^c^ The category for other was not further defined in the database; 1103 (35.9%) of patients in this group had unknown race or ethnicity.

Approximately 11.7% (n = 3581) of adolescents screened positive for depressive symptoms and 2.7% (n = 892) screened positive for suicidality at any well-teen visit over the study period. Approximately 30.8% of adolescents (n = 10 559) had completed more than 1 screening. Screening rates were similar across ICE quintiles and race and ethnicity. Depressive symptoms were most prevalent (both 12.5%) among Black (n = 322) and Hispanic (n = 1099) adolescents and least prevalent (8.6%) among Asian or Pacific Islander adolescents (n = 620). Similarly, suicidality was highest (4.0%) among Black adolescents (n = 102) and lowest (1.9%) in Asian or Pacific Islander adolescents (n = 139).

There were significant associations between the ICE and adolescent depressive symptoms and suicidality (Table 2). There were general dose-response relationships for ICE–income and ICE–income plus race, whereby each level of declining neighborhood privilege was associated with greater risk of depressive symptoms. Controlling for sex, race and ethnicity, maternal education level, and maternal age at delivery, the ARR for depressive symptoms comparing the least- (quintile 1) vs most-privileged (quintile 5) quintiles were 1.22 (95% CI, 1.08-1.38) for ICE–race, 1.26 (95% CI, 1.11-1.44) for ICE–income, and 1.37 (95% CI, 1.20-1.55) for ICE–income plus race. For suicidality, a dose-response pattern was observed for ICE–income, with ARRs of 1.21 (95% CI, 1.01-1.45) in quintile 4 and 1.59 (95% CI, 1.24-2.05) in quintile 1. ICE–race was not significantly associated with suicidality. For ICE–income plus race, the ARR for suicidality comparing quintile 1 vs quintile 5 was 1.59 (95% CI, 1.23-2.05), while risks for quintiles 2 to 4 were not significantly different.

**Table 2.** Associations Between the ICE and Depressive Symptoms and Suicidality Among Adolescents Aged 12 to 16 Years, Unadjusted and Adjusted for Individual-Level Sociodemographic Characteristics

ICE measure	Depressive symptoms		Suicidality	
	RR (95% CI)	ARR (95% CI)^a^	RR (95% CI)	ARR (95% CI)^a^
**ICE–race and ethnicity**				
Quintile 5 (most privileged)	1 [Reference]	1 [Reference]	1 [Reference]	1 [Reference]
Quintile 4	1.07 (0.96-1.20)	1.07 (0.96-1.20)	0.97 (0.78-1.21)	0.96 (0.77-1.20)
Quintile 3	1.07 (0.96-1.20)	1.06 (0.95-1.19)	0.86 (0.69-1.08)	0.85 (0.68-1.08)
Quintile 2	1.19 (1.07-1.33)	1.16 (1.04-1.30)	0.90 (0.72-1.12)	0.86 (0.68-1.08)
Quintile 1 (least privileged)	1.32 (1.17-1.48)	1.22 (1.08-1.38)	1.02 (0.81-1.29)	0.90 (0.70-1.17)
**ICE–income**				
Quintile 5 (most privileged)	1 [Reference]	1 [Reference]	1 [Reference]	1 [Reference]
Quintile 4	1.13 (1.04-1.23)	1.06 (0.98-1.16)	1.28 (1.07-1.52)	1.21 (1.01-1.45)
Quintile 3	1.19 (1.09-1.30)	1.09 (0.99-1.19)	1.30 (1.08-1.58)	1.20 (0.98-1.45)
Quintile 2	1.39 (1.26-1.54)	1.25 (1.13-1.39)	1.42 (1.14-1.77)	1.27 (1.01-1.60)
Quintile 1 (least privileged)	1.44 (1.27-1.63)	1.26 (1.11-1.44)	1.83 (1.44-2.32)	1.59 (1.24-2.05)
**ICE–income plus race and ethnicity**				
Quintile 5 (most privileged)	1 [Reference]	1 [Reference]	1 [Reference]	1 [Reference]
Quintile 4	1.08 (0.98-1.18)	1.06 (0.97-1.16)	1.07 (0.89-1.29)	1.07 (0.89-1.29)
Quintile 3	1.18 (1.08-1.29)	1.13 (1.03-1.24)	1.11 (0.92-1.34)	1.07 (0.88-1.30)
Quintile 2	1.42 (1.28-1.56)	1.29 (1.17-1.43)	1.16 (0.92-1.45)	1.06 (0.84-1.35)
Quintile 1 (least privileged)	1.51 (1.34-1.71)	1.37 (1.20-1.55)	1.79 (1.41-2.26)	1.59 (1.23-2.05)

Abbreviations: ARR, adjusted risk ratio; ICE, Index of Concentration at the Extremes.

^a^ Adjusted model covariates include adolescent sex, adolescent race and ethnicity, maternal education level, and maternal age at delivery.

Greater risks of depressive symptoms and suicidality persisted among certain racial and ethnic groups after adjusting for individual-level covariates. Compared to White youth, Asian youth showed lower risks of both outcomes (depressive symptoms: ARR, 0.87, 95% CI, 0.79-0.96; suicidality: ARR, 0.79; 95% CI, 0.63-0.98).

Black youth had higher risks than White youth, with ARRs of 1.21 (95% CI, 1.08-1.36) for depressive symptoms and 1.42 (95% CI, 1.13-1.78) for suicidality. Hispanic youth also had higher risks of depressive symptoms (ARR, 1.13; 95% CI, 1.04-1.23) as White youth but a similar risk of suicidality (ARR, 0.92; 95% CI, 0.77-1.10). The ARRs comparing other or unknown youth with White youth were not significant for either outcome (depressive symptoms: 1.13 [ 95% CI, 0.90-1.42]; suicidality: 1.01 [95% CI, 0.90-1.13]). We also observed racial and ethnic disparities in depressive symptoms before and after adjusting for ICE measures (Table 3). The depressive symptom disparities between Black and White youth, as well as Hispanic and White youth, decreased after adjusting for each ICE measure, and became nonsignificant in models adjusting for ICE–race and ICE–income plus race. The Black-White disparity in suicidality reduced from an ARR of 1.42 (95% CI, 1.13-1.78) unadjusted for ICE to ARRs of 1.31 (95% CI, 1.03-1.66) and 1.30 (95% CI, 1.01-1.67) after adjusting for ICE–income and ICE–income plus race, respectively.

**Table 3.** Racial and Ethnic Disparities in Depressive Symptoms and Suicidality Among Adolescents Aged 12 to 16 years, Unadjusted and Adjusted for ICE

Model^a^	RR (95% CI)		
	Black-White disparity		Hispanic-White disparity, depressive symptoms
	Depressive symptoms	Suicidality	
1. Unadjusted for ICE	1.21 (1.08-1.36)	1.42 (1.13-1.78)	1.13 (1.04-1.23)
2. Adjusted for ICE–race and ethnicity	1.11 (0.97-1.26)	1.46 (1.12-1.89)	1.08 (0.99-1.18)
3. Adjusted for ICE–income	1.14 (1.01-1.29)	1.31 (1.03-1.66)	1.11 (1.02-1.21)
4. Adjusted for ICE–income plus race and ethnicity	1.10 (0.97-1.24)	1.30 (1.01-1.67)	1.08 (0.99-1.18)

Abbreviations: ICE, Index of Concentration at the Extremes; RR, risk ratio.

^a^ All models adjusted for adolescent sex, adolescent race and ethnicity, maternal education level, and maternal age at delivery.

## Discussion

In this large, racially and ethnically diverse cohort of adolescents, those from neighborhoods with extreme concentrations of racial and economic disadvantage were more likely to screen positive for depressive symptoms and suicidality at well-teen visits compared to their counterparts from the most racially and economically privileged neighborhoods. Consistent with our hypotheses, these neighborhood-level disparities remained even after accounting for individual-level sociodemographic characteristics, and adjustment for these measures of neighborhood economic and racial inequality (ICE) revealed reduced racial and ethnic disparities within the cohort.

To our knowledge, this is the first study to assess the impact of combined neighborhood racial and economic inequality, as captured by ICE, on mental health outcomes.^39^ Our study revealed clear gradient relationships, whereby adolescents had progressively higher risks of depressive symptoms and suicidality as levels of neighborhood disadvantage increased. The combined measure of neighborhood racial and economic privilege (ICE–income plus race) was associated with greater risk of adolescent depressive symptoms compared with the separate measures of ICE–race and ICE–income. In contrast, ICE–income plus race and ICE–income both were associated with suicidality, while ICE–race alone did not show a significant association. Despite the large sample size, suicidality was a rare outcome, which may have limited the statistical power to detect significant associations. Overall, our findings suggest that neighborhood racial and economic privilege contribute to mental health disparities among adolescents. The stronger effect size observed for ICE–income plus race vis-à-vis depressive symptoms aligns with most epidemiologic studies using ICE measures.^39^ Our findings support the notion that the combined measure of racial and economic neighborhood privilege may better capture the social dynamics giving rise to health inequities than measures that examine these dimensions separately.^24,25^

Our study extends the small body of research on structural racism and mental health outcomes. Previous ecological studies found higher rates of suicide among young Black males in cities with larger occupational and economic disparities between Black and White residents,^54,55^ as well as in cities with greater disadvantage among Black males, as measured by unemployment, education, income, and racial residential segregation.^55,56^ Additionally, a recent study^30^ linked structural racism, as measured by historical redlining and present-day neighborhood lending discrimination, with poorer mental health in adult residents. Sugg et al^57^ found associations between ICE–race and ICE–income measures and spatial clusters of youth suicide and self-injury in North Carolina but did not use the combined ICE–income plus race measure. In contrast to these ecological designs, our study used outcome data measured at the individual level, allowing for the inclusion of individual-level sociodemographic characteristics in the analysis. The persistence of associations between ICE and adolescent depressive symptoms and suicidality even after controlling for individual-level sociodemographic factors suggests that the impact of neighborhood racial and economic inequality goes beyond the individual characteristics of adolescents, highlighting a likely influence of contextual-level features of neighborhoods on mental health outcomes. The present study’s focus on adolescents aged 12 to 16 is also important, given that 50% of all lifetime cases of mental health disorders begin by age 14^58^ and mental health disparities are widening in this age group.^5,59,60^

Whereas most research on racial residential segregation and mental health has focused on 1 racially and ethnically minoritized group (eg, Black individuals),^61,62,63^ our study is unique in that it examines the joint associations of racial and economic inequality with mental health outcomes across a diverse cohort of adolescents. Our findings suggest that structural racism permeates society and affects the mental health of adolescents from various racial and ethnic backgrounds. Moreover, by capturing the relative distribution of resources within a neighborhood, use of the ICE measure moves beyond reductive approaches that focus exclusively on the “disadvantaged” to a more critical framing of health inequities as arising from unequal distributions of power, resources, and opportunity rooted in White privilege and anti-Black policies and practices.^37^ While we calculated ICE measures for racial privilege using Black-White comparisons, it is important for future research to examine other racial and ethnic dichotomies using the ICE measure. Understanding the joint significance of racial and economic inequality across different groups will provide a more comprehensive understanding of the dynamics of structural racism and its associations with adolescent mental health. In addition, qualitative research focused on the lived experiences of adolescents in structurally disadvantaged neighborhoods would afford greater insight into the associations of historically racist practices and policies with individual mental health.

### Limitations

This study has limitations. First, the patients in the study cohort were insured and presenting for well-teen visits, limiting generalizability to uninsured or otherwise medically underserved adolescents. Specifically, use of the KPNC population may have led to more conservative estimates than what would be observed in a community sample, given the underrepresentation of individuals with very low income in the health system.^42^ Second, we were restricted to sociodemographic information documented in the EHR and thus residual confounding from unmeasured socioeconomic variables (eg, household income) cannot be ruled out. Third, outcome measures were based on brief self-report screeners in primary care rather than clinical interviews. While this reduced potential for selection bias and the PHQ-2 has good sensitivity and specificity for detecting major depression in adolescents,^45^ single-item measures of suicidality have high levels of measurement error, including both under- and overreporting.^64,65^ In particular, the use of the word “seriously” in our suicidality measure may have resulted in underreporting of less severe but more prevalent forms of suicidality.^66^ Fourth, due to small numbers in our sample, we combined American Indian or Indigenous adolescents with those in the category of other or unknown. Additionally, the database we used had already aggregated Pacific Islander and Asian youth into a single racial category. As a result, we were unable to examine impacts of ICE in these specific subgroups of adolescents who may have disproportionately high rates of depression and suicidality.^67^

## Conclusions

This cohort study identified neighborhood racial and economic privilege as independent factors associated with adolescent depressive symptoms and suicidality in a socioeconomically and racially and ethnically diverse population. These findings support the utility of using ICE to examine mental health disparities at the census-tract level. These findings also suggest that interventions aimed at promoting equity in adolescent mental health outcomes must extend beyond the clinical setting and engage more directly with the economic, social, and political structures that perpetuate inequitable distributions of resources across neighborhoods.

## Notes

Supplement.
Data Sharing Statement
