# Supplementary material for: Structural Racism and Adolescent Mental Health Disparities in Northern California
Source: JAMA Netw Open. 2023 Aug 18;6(8):e2329825. doi: 10.1001/jamanetworkopen.2023.29825 (PMC10439477; doi:10.1001/jamanetworkopen.2023.29825)
Supplement: Supplement. — Data Sharing Statement [file jamanetwopen-e2329825-s001.pdf]

Data Sharing Statement

Acker. Structural Racism and Adolescent Mental Health Disparities in Northern California. *JAMA Netw Open*. Published August 18, 2023. doi:10.1001/jamanetworkopen.2023.29825

Data
Data available: No

Additional Information
Explanation for why data not available: The datasets generated and/or analyzed during the current study are not publicly available due to our institutional policy. Individuals who are interested in accessing the data may contact the corresponding author regarding [or to discuss or set up] a data use agreement
